# RCT evaluation of Skhokho: A holistic school intervention to prevent gender-based violence among South African Grade 8s

**DOI:** 10.1371/journal.pone.0223562

**Published:** 2019-10-29

**Authors:** Rachel Jewkes, Anik Gevers, Esnat Chirwa, Pinky Mahlangu, Simukai Shamu, Nwabisa Shai, Carl Lombard

**Affiliations:** 1 Gender & Health Research Unit, South African Medical Research Council, Pretoria, South Africa; 2 School of Public Health, Faculty of Health Sciences, University of the Witwatersrand, Johannesburg, South Africa; 3 Independent Consultant, Cape Town, South Africa; 4 Foundation for Professional Development, Pretoria, South Africa; 5 Biostatistics Research Unit, South African Medical Research Council, Cape Town, South Africa; Arizona State University, UNITED STATES

## Abstract

We evaluated the effectiveness of the Skhokho interventions (enhanced teaching materials and a parenting programme) in reducing dating and sexual violence. This pragmatic, three-arm cluster randomised controlled trial was conducted in 24 State schools in Grade 8 classes, with all learners invited to participate. The interventions were: i) a schools’ package: A Life Orientation (LO) curriculum workbook for the Grade 8 national curriculum and teacher training; ii) a workshop for caregivers and teenagers, supported by clubs in the second year. Arms were: a no intervention control, the schools’ package, and the combined schools’ and families’ package. Learners were followed up for 18 months. The primary outcomes were the incidence of physical and/or sexual IPV, severe IPV and non-partner rape. At baseline, 3756 (61.8% of total) learners (aged 12–15) were interviewed and 3411 (90.8%) provided end line data. A third of caregivers and learners attended all four families’ intervention workshop sessions. At baseline, 47% of girls and 29% boys were dating and 18% of boys and 2% of girls had had sex. Differences in the primary outcomes between study arms were not statistically significant, however all effects were in the direction of protection from violence and several secondary outcomes were significantly changed. For girls, the incidence of any IPV experience was aIRR 0.84 (95%CI 0.66, 1.07 p = 0.159) for the school’s arm and the incidence of non-partner rape was aIRR 0.84 (95%CI 0.62, 1.14 p = 0.255) for the combined schools and families arm v. control arm. This under-powered pragmatic study’s findings suggest a generally beneficial impact of the Skhokho interventions on a number of outcome measures, when viewed by both adolescents, caregivers and their teachers. These include measures of adolescents’ exposure to violence, improved sexual health and reductions in several IPV risk factors. The intervention warrants further research.

**Trial registration:**

ClinicalTrials.gov NCT02349321.

## Introduction

Rape and intimate partner violence (IPV) are highly prevalent in South Africa [1, 2]. Perpetration generally commences in the teenage years, with IPV commonly a feature of early dating relationships [3, 4]. Most men who perpetrate rape do so for the first time as teenagers [5]. IPV and sexual violence undermine teenager girls’ school completion, due to their impact on health generally and particularly teenage pregnancy [6]. Research conducted with school students in the rural Eastern Cape province of South Africa found that 26.5% of girls in grade 10 had experienced more than one episode of physical or sexual IPV and 5.6% had been raped by a non-partner[7], and 31.8% of boys in grade 9–10 disclosed perpetration of physical and or sexual IPV and 16.3% had raped a non-partner or participated in gang rape [8, 9]. Education itself seems to reduce women’s risk of IPV and men’s risk of perpetration [10, 11], but its impact on sexual violence risk is less clear [12, 13].

Developing interventions to prevent and enable responses to dating and sexual violence experienced by teenagers is a very high priority for violence prevention. The school setting potentially enables interventions to be delivered before dating or other sexual violence has been experienced or perpetrated and thus potentially enabled foundational beliefs, gender attitudes and behaviours to be changed. Almost all children attend school at some stage and mostly are retained in the system to grade 8, so there is a great possibility for access to the population at an impressionable age through schools. Schools are thus a critical setting and provide a valuable potential platform for intervention delivery and scale up.

Despite the strong rationale, schools are a challenging environment for effective sexual relationship and health intervention delivery. Most of the multitude of HIV prevention initiatives developed and tested in schools in South Africa and other Sub-Saharan African countries have not changed behaviour [14–16]. Interventions that have used the school setting and have been effective in prevention of partner violence have tended to use out of school delivery opportunities, and two notable examples with positive findings on some violence prevention measures, Prepare and Stepping Stones, were evaluated in South Africa [17, 18]. There has also been effect shown with the use of participatory methods and curriculum during class time, as seen with the Fourth R intervention in Canada [19], and there may have been impact from a building intervention in Shifting Boundaries in New York, although the lack of a consistent result found in the building plus classroom arm raises questions about whether the building only result may have been due to chance [20]. An exception was the ‘Good Schools’ the participatory, whole school intervention in Uganda, which was shown to effect a reduction in corporal punishment at school [21]. Several other evaluations of interventions in the classroom space in schools conducted north America have not shown positive results on preventing physical or sexual dating violence [22–29].

There is a good argument to make for involving caregivers in violence prevention and the potential of parenting interventions for teenagers to enable protection in this regard. However, in low- and middle-income countries there has not been a great deal of research developing and evaluating such interventions. A recent randomised controlled trial from Thailand found a parenting programme improved relationship quality, less harsh discipline and better family cohesion [30], and an intervention, Sinovuyo, evaluated in South Africa among families reporting conflict and 5–9 months after the intervention found caregivers and adolescents reported less poor supervision and more involved parenting, and caregivers reported several other positive outcomes [31]. These interventions did not examine impact on adolescents’ risk of violence. Two systematic reviews of parenting programmes have failed to identity other evaluated programmes for caregivers of adolescents [32, 33].

In South Africa, the context for scalable violence prevention in State schools is framed by the national curriculum. This includes a lesson of Life Orientation (often called LO), taught in all grades, that covers relationships, health, citizenship and preparation for careers and work. Its content includes gender equity and violence prevention. In 2011 when this research was conceptualised, there was a Curriculum and Assessment Policy Statement (CAPS) for LO which stated the topics, session objectives and duration for the entire school year per grade, but teaching materials were not specified. Life Orientation provided a very positive opportunity for intervention to prevent violence, but schools also have many problems. South African education has been scarred by the apartheid policy of Bantu Education, through which Black Africans were denied decent education. Two decades after the end of apartheid, the country still struggles with this legacy, and educational outcomes are unacceptably poor and between 60–80% of schools are viewed by educationalists as dysfunctional [34]. Many teachers are not able to teach, and engage in anti-social activities, including use of physical and sexual violence against students, often with protection from their trade unions [34]. Corporal punishment in school was banned in 1996 but in 2012 half of all students in a national survey disclosed having experienced it [35]. The tolerance of authorities to its continuation is explained in terms of classroom discipline, which is an acknowledged major problem [36]. Hearings on school violence of the South African Human Rights Commission have shown that schools are often the place where students feel least safe [37].

Educationalists have considered the question of how South African schools can be made into empowering transformative spaces and argue that to do these three key players in a school need to be engaged. These are teachers, students and the caregivers [38]. Furthermore, success in changing gender norms in schools, requires the availability of interventions: i.e. an appropriate curriculum, student support services, policies to strengthen the institution, and attention to interactions in the school, for example the gender climate and the interpersonal aspects of teaching. An example of the latter would be training teachers in how to teach and addressing their values and attitudes as well as training them in what to teach [38].

**Fig 1:**
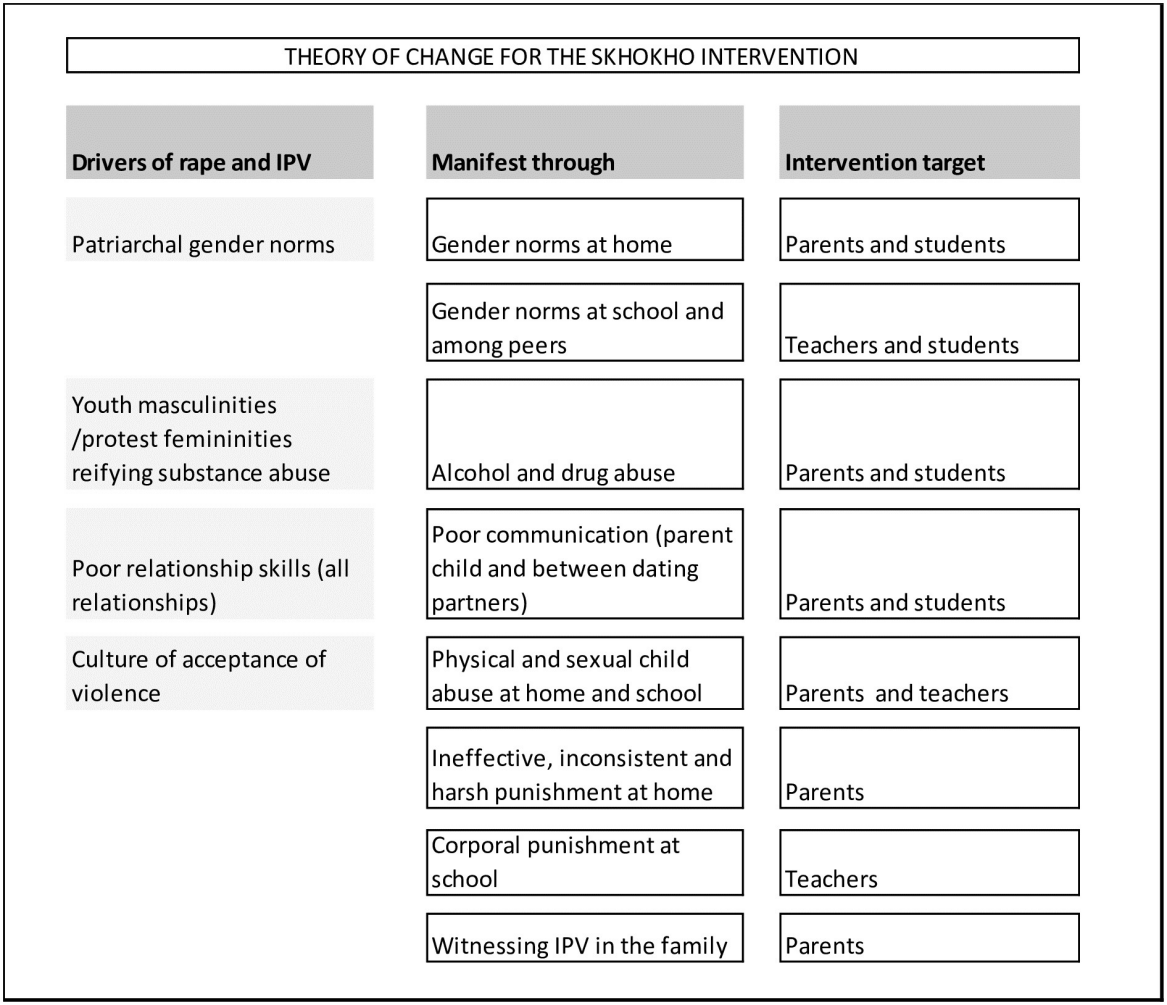
Theory of change for the Skhokho intervention.

The Skhokho intervention package was developed with the goal of addressing the drivers of rape and IPV among adolescents [39, 40]. We considered how they were manifest in the adolescents’ social environment and thence how we should intervene on them, as shown in the Theory of Change (Fig 1). Subsequent to the start of the study we analysed baseline data on IPV risk factors and these largely confirm the theory of change [41]. Drawing on the education theory summarised by Morrell and the framework of the national LO curriculum, we developed interventions to impact the manifestations of the drivers of IPV and rape. This paper presents the results of a randomised controlled trial which was conducted to evaluate the impact of the Skhokho intervention in schools in Tshwane metropole, South Africa. We hypothesised that Grade 8 learners exposed to the schools intervention would have a lower incidence of IPV and non-partner rape experience (girls) and perpetration (boys) and that those who had the combined schools and families interventions would have lower incidence still.

## Methods

The study was a cluster randomised trial, with the cluster design chosen because the intervention is delivered to grades in schools. It was set in high schools among the Grade 8 year, located in the environs of Pretoria (the metropolitan area of Tshwane) and the unit of randomisation was a high school. We compared three study arms in the trial: there was a control arm, which was essentially business as usual within the eight randomly selected schools; the other two arms both receiving the schools intervention package, and in one of these arms, caregivers were invited to attend a 4 day workshop with their child on parent teenager relationships (the “families” arm).

### Interventions

**Fig 2:**
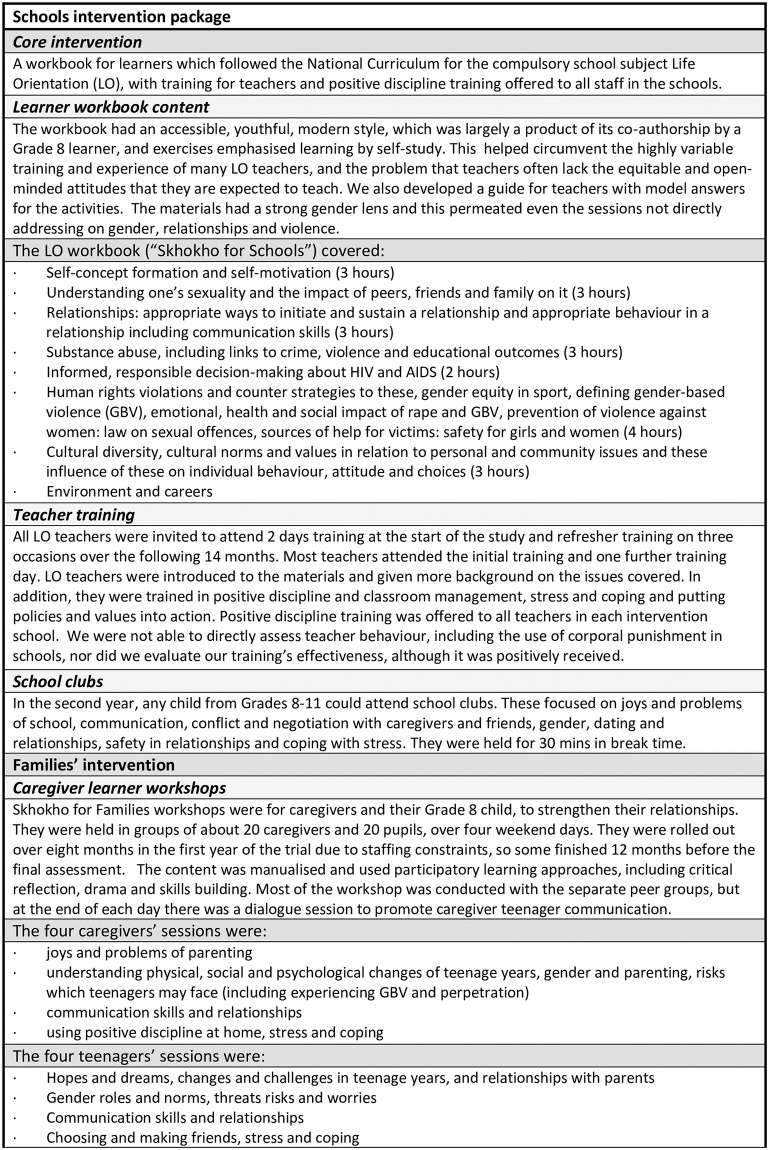
Intervention panel.

Thus, both the interventions arms received the schools’ intervention, and in addition one arm received the family’s intervention. These are described in the intervention panel (Fig 2). All of the children in Grade 8 should have received the schools’ intervention, irrespective of study participation, and all those in the families’ arm were invited to the workshop. Our study was a pragmatic trial, with all of the interventions implemented as if in broader community roll-out. The LO teacher training and positive discipline sessions were facilitated by project staff. The schools’ normal Life Orientation (LO) teachers delivered the LO lessons. Because the LO intervention was delivered in regular school classes, we do not have information about attendance, but we were able to ascertain that the workbook was used in each school. The standard LO materials were also available to teachers, and some had materials from a range of other sources, and so we could not verify fidelity to the workbook.

The workshops’ and school clubs were facilitated by pairs of project staff (mostly female), who were employed, trained and supervised by the SAMRC. The workshop facilitators were paired according to their age, to ensure that older facilitators conducted the caregivers’ workshops and the younger facilitators conducting the teen’s sessions. The sessions were held on school premises at weekend. Caregivers and teenagers were provided with tea and lunch in the workshop, and a light snack in the school clubs. The caregivers participating in the workshops were given R50, and teenagers given R20 at the end of each session to reimburse the cost of transport used to attend the workshop as most of them were staying far from the school.

### Research design

The schools constituted twenty-four clusters. Eligible high schools were approximately 3 km from the nearest school (to minimise contamination of study arms) and were willing to participate (established through a process of school mobilisation). They were State secondary schools that taught in English that were within 50km of the SAMRC office in Pretoria. We excluded former model C schools (high fee-paying, elite State schools) and schools teaching in Afrikaans because of the heterogeneity they would have introduced, and the financial and operational complexity of making available classroom materials in two languages. The schools were grouped into three geographical strata and equal numbers of clusters were allocated to each arm, within each stratum. The balanced allocation sequence for each stratum was generated by the study statistician (CL) based in Cape Town using a table of random digits, who had no particular knowledge of schools in the study area. Schools were recruited prior to randomisation. The clusters were identified and randomised at the end of one academic year and soon after the start of the next the participants were enrolled. There was no blinding as for logistical reasons and randomisation was done before individual study participant recruitment.

In each school we sought to recruit the entire Grade 8 and enrol them in a closed cohort for the study. Schools were mixed sex and the 8^th^ Grade ranged from 140–442 students. Recruitment started with a general meeting of the caregivers and pupils where the study was explained. Pupils were then given consent forms, the study brochure and information sheets, and asked to take them home to their caregivers, with a request that they be signed to indicate if caregivers did or did not consent to their child’s participation. To incentivise return of the forms, we held a raffle in each school, with raffle entry for those who had returned a caregiver consent form (whether agreeing or otherwise to participate). We made a video explaining about the study and one to explain about the raffle.

All children who returned caregiver consent forms and agreed to participate went through a formal consent procedure where a project staff member read aloud the study’s consent form to the class and gave an opportunity for questions. Informed consent forms were given to the children and they were invited to sign. We paid no incentives for intervention attendance nor to children for interviews.

### Sample size

The sample size, i.e. the number of clusters required in each arm, was calculated using the method of Hayes and Bennett [42]. The calculation assumed that the effect (as measured by incidence rate ratio) would be homogeneous for males and females, and assumed a 12 month cumulative IPV incidence rate (averaged over males and females) in the control arm of 10% and that 18 month interviews providing data for incidence results would be obtained assuming 200 person years of follow up per school (133 students followed up for 18 months). The 10% estimate was an extrapolation from research from Cape Town with the same age group where physical IPV perpetration (reports by boys) and victimisation (by girls) in the past 3 months among Grade 8 students was found to be 3.9% and 6.6% respectively [43]. We anticipated 250 students per grade enrolling in the study and that allowed for about 20% loss to follow up, or fewer initially recruited. The sample size calculation required an estimate of k, the between cluster coefficient of variation of the outcome measure; we used k = 0.20. A sample size of 8 clusters per trial arm would then give more than 80% power to detect as statistically significant at the 5% level a 50% reduction in IPV incidence. This level of incidence reduction has been previously achieved in IPV prevention intervention evaluation[44], but was chiefly chosen to match the sample size with available resources.

Questionnaires were administered in class after the school teaching day, and prior to the start of the family’s intervention (baseline), and after approximately 6, 12 and 18 months. Completion took about an hour. The baseline interviews were staggered over a three-month period (February—April 2014) and this was after the start of the LO curriculum (taught from mid-January). The first follow up round (which we call 6 months) was between August and September 2014, the 12 months interviews were held from (February—April 2015) and end-line interviews (which we refer to as 18 months follow up) were from August–September 2015. Participants were located for repeat interviews using details collected at enrolment. If they had moved within the study area, we tried to find them in their new location, or at home. We tracked participants ID numbers to enable linking of different rounds of interviews with an electronic thumb print reader.

The researchers worked closely with the National Department of Basic Education’s Social Cohesion and Equity Unit and gained support from the Director General of the Department of Basic Education. Permission to work in Gauteng Schools was given by the Gauteng Department of Education, and letters were provided by the Tshwane district offices. The SAMRC Ethics Committee gave approval for the study. We had a community advisory board comprising teachers, educational officials, national schools governing bodies associated representatives, Principals, some caregivers and learners. Consent for the research was signed by caregivers and by learners (as described above) but all interventions were offered to all pupils (whether or not consented for and involved in the research). All children in the class were given a pocket booklet with phone numbers for help for a range of different problems from violence to substance abuse. Any child who specifically identified him or herself as in need of help during the study period was taken by teachers to appropriate services, as per standard practice.

### Questionnaire

The impact of the intervention on behaviour and attitudes was assessed with a questionnaire self-administered in Sepedi or Setswana or English on an iPod touch platform. The learners listened to the questions on headphones and read them on the screen. The outcome measures, indicators and assessment are described in Table 1. We had initially intended to have another secondary outcome on the incidence of emotional abuse (captured by 3 questions) but we did not pursue this as the incidence was too high to be meaningful as a study outcome. Where we developed our own measures, we first reviewed other measures/scales on related topics, then as a study team went through and adapted where we thought necessary based on our previous experience doing research with similar populations in South Africa, then we piloted the questionnaire with a few pupils to ensure that items were understood.

**Table 1:** Table of learners’ measures.

Construct	Measure	Typical item	Cronbach's alpha	Source	Expected change
**Primary outcome**					
Incidence of IPV	Experience of (girls) or perpetration of (boys) any physical or sexual IPV	"in the past 12 months has a boyfriend slapped you or thrown something at you with could hurt you?		Garcia-Moreno et al 2005; Jewkes at al 2011; lightly adapted	↓
Incidence of severe IPV	An affirmative response to more than one physical or sexual IPV question at 12 or 18 months. Each question starting "in the last 12 months…"	"have you had sex with a boyfriend when you didn't want to because he forced or threatened or pressurised you or because you were unable to stop him?"		Garcia-Moreno et al 2005; Jewkes at al 2011; lightly adapted for the age group	↓
Incidence of non-partner rape	two/three items ask about single perpetrator rape, multiple perpetrator (asked of boys only) and rape when drugged or drunk. Response categories for boys ask if it was in the last 6 months, for girls the frequency ever	Have you and other boys had sex with a woman at the same time when you forced her or you tricked her?		Jewkes at al 2011;	↓
**Secondary outcomes**					
Caregiver child communication scores (comparison of arms at 18m)	Five items with a Likert scale response (high score = more communication)	"How often does one of your parents or caretakers ask about your day or how was school?"	boys = 0.655 girls = 0.678	developed for the study	↑
Childhood trauma scores (comparison of arms at 18m)	13 items with a 3-level response: 1 = never, 2 = between 6–12m, 3 = in last 6m,	"I have been insulted or humiliated by someone in my family in front of other people"	boys = 0.693 girls = 0.628	adapted form Bernstein et al 2003	↓
Bulling at school score (comparison of arms at 18m)	Three items with a 4 point Likert response scale (strongly agree, agree, disagree, strongly disagree)	"I like to make fun of others at school, especially the weak ones"	boys = 0.688 girls = 0.675	developed for the study	↓
Communication with a girlfriend or boyfriend (comparison of arms at 18m)	Three items with a 4 point Likert response scale (very often, sometimes, seldom, not at all)	" in the past month, how often do you and your girlfriend or boyfriend discuss your hopes and dreams?"	boys = 0.713 girls = 0.763	developed for the study	↑
Delinquency score (comparison of arms at 18m)	Five items	" How often have you been involved in a fight with knives?"	boys = 0.766 girls = 0.684	adapted from Tremblay 1995	↓
Depression score (comparison of arms at 18m)	Twenty two items asking about the past 2 weeks starting "which of the sentences best described your thoughts and feelings in the last 2 weeks?"	"I get along with people, I get into fights many times, I get into fights all the time"		Kovacs, 1992; adapted for South Africa	↓
Gender attitudes score (comparison of arms at 18m)	Five items. High = more gender equitable	" I think there are times when a woman deserves to be beaten"	boys = 0.595 girls = 0.552	Jewkes 2002, adapted	↑

Study participants completing the 12- and 18-month interview were asked a question about how honest they were in completing the questionnaire, with five response categories between ‘completely honest’ and ‘not honest at all’. We classified a person as ‘honest’ if they reported having been ‘fairly, very or completely’ honest at their last interview.

### Interviews with caregivers and teachers

Interviews were also conducted with the caregivers who indicated an intention to attend the family’s intervention and with teachers from the schools. We hypothesised that the intervention might reduce IPV experienced by female caregivers, as well as reduce patriarchal gender attitudes and strengthen measures of parenting and communication with the child.

The caregivers research was a modified interrupted time series. Caregivers were recruited during the school meetings, and those who agreed to participate were requested to come to school on an agreed Saturday. The first two Saturdays were used for data collection (two baselines) and then the workshops commenced. Caregivers gave written consent to participate in the research. the questionnaire was self-administered on an audio-assisted PDA in English, Sepedi or Setswana. They were invited to complete the same questionnaire on three further occasions: 6 months after baseline, 12 months and 18 months later. The caregivers interviewed at the follow up points were located through their addresses and phone numbers, and largely interviewed at home. The analysis followed intention to treat.

To recruit teachers, project staff went to the staff room during lunchbreak. They started informally chatting about the research and to those who indicated interest were invited to consent to the interview. The teachers interviewed were an open cohort, that is at baseline, 12 months and then 18 months those interviewed were dependant on who was available and willing. An incentive of R50 (less than $4) was given after each parent and teacher interview. The main measures used in the caregivers’ and teachers’ questionnaires are presented in Table 2. All participants including learners, teachers and caregivers were given an opportunity to withdraw and refuse an interview at any data collection point.

**Table 2:** Table of caregivers’ and teachers’ measures.

Construct	Measure	Typical item	Cronbach's alpha	Source	Expected change
**Women parents' experience of intimate partner violence**					
IPV	Emotional IPV in last 6 months (2 items asking about 'in the past 6 months has your husband or a boyfriend …')	ever threatened to hurt you? ever not provided money to run the house or look after the children, but has money for other things?		Garcia-Moreno et al 2005 adapted;	↓
	Physical IPV ever (3 items asking about 'Has your husband or a boyfriend ever …') followed by one question: Have you been slapped, beaten or hurt in any of these ways in the past 6 months?	slapped you, pushed or shoved you, or threw something at you which could hurt you? hit you with a fist or with something else which could hurt you? kicked, dragged, choked or burnt you or threatened to use or actually use a gun, knife or other weapon against you?		Garcia-Moreno et al 2005 adapted;	↓
	Sexual IPV ever: 2 items, each followed by one question: was it in the past 6 months?	physically forced you to have sex when you did not want to? Did you ever have sexual intercourse when you did not want to because you were afraid of what your partner might do?		Garcia-Moreno et al 2005 adapted;	↓
	Physical or sexual IPV in the lifetime	any reports of physical or sexual IPV in the above questions			↓
	Any IPV in the last 6 months	any emotional, physical or sexual IPV reported in the above questions			↓
**Other outcomes**					
Caregiver child communication scores	5 items with a responses every day, each week, at least once a month, sometimes but not each month, never	How often do you ask your Grade 8 child how was his or her day or how was school?	Male = 0.83 Female = 0.77	developed for the study	↑
Knowledge of the child	13 items: 4 point Lickert scale responses strongly agree, to strongly disagree	I know about my Grade 8 child’s future dreams and plans	Male = 0.85 Female = 0.87	developed for the study	↑
Stress score	13 items Compassion Fatigue Scale-Revised, adapted as a child raising fatigue scale: 5 point Likert response scale (not at all to very often)	I have felt a sense of hopelessness about my child or children	Male = 0.87 Female = 0.90	Gentry et al 2002	↓
Positive parenting score	29 items on authoritative, supportive parenting and positive discipline with involvement and monitoring at home: 4 point Lickert scale responses strongly agree, to strongly disagree	I encourage my Grade 8 child always to do his/her best	Male = 0.91 Female = 0.92	(from Block's Childrearing practices report modified, Rickel & Biasatti 1982)	↓
Negative parenting score	7 items on neglectful parenting and negative discipline: 4 point Lickert scale responses strongly agree, to strongly disagree	I believe physical punishment to be the best way of disciplining	Male = 0.73 Female = 0.71	(Rickel & Biasatti 1982)	↓
Childhood trauma score	8 items (6 emotional abuse/neglect, 2 physical abuse) with response categories: in the last 6 months, between 6–12 months, before the last 12 months, never	I have been too drunk to care for my Grade 8 child	Male = 0.56 Female = 0.66	adapted from Bernstein 2003	↓
Individual gender attitudes score	9 items, 4 point Lickert scale responses strongly agree, to strongly disagree. High = more gender equitable	I think a man should have the final say in family matters.	Male = 0.79 Female = 0.76	Jewkes 2002, adapted	↑
Social norms on gender score	9 items, 4 point Lickert scale responses strongly agree, to strongly disagree. High = more gender equitable	My community thinks that if a girl dresses sexy or gets drunk she’s inviting men to rape her	Male = 0.88 Female = 0.83	Jewkes 2002, adapted	↑
General health	16 items, yes/no responses, summed	Do you often have headaches	Male = 0.89 Female = 0.89		↑
Embarrassed at talking about sex with child	One item: responses very, somewhat, not at all	If your grade 8 child wanted to talk about sex with you, how embarrassed would you be?		developed for study	↓
Offended by talking about sex with child	One item: responses very, somewhat, not at all	If your Grade 8 child wanted to talk about sex with you how offended would you be?		developed for study	↓
**Measures for teachers' questionnaire**					
Perception of school environment	18 items: 4 point Lickert scale responses strongly agree, to strongly disagree	Our school has a sense of vision, and a mission that is recognised by all staff and learners	0.89	developed for study	↑
Bulling in school	11 items: 4 point Lickert scale responses strongly agree, to strongly disagree	Learners may be bullied at school if they are thought to be gay or lesbian.	0.88	developed for study	↓
Teachers' negative behaviour	9 items: 4 point Lickert scale responses strongly agree, to strongly disagree	Teachers often flirt with the learners at our school.	0.80	developed for study	↓
Work stress	13 items based on the Compassion Fatigue Scale-Revised, adapted as a child raising fatigue scale: 5 point Likert response scale (not at all to very often)	I have experienced troubling dreams about my work.	0.92	Gentry et al 2002	↓
Perpetration of corporal punishment	1 item: yes/no	In the past 6 months I beat a learner or sent one to another teacher for beating.		developed for study	↓

### Data analysis

The analysis was intention to treat and thus included all learners for whom we had baseline and end line data but excluded those lost to follow-up at 12 and 18 months. We looked for any association between loss to follow up and intervention arm. Analysis of the primary outcomes (IPV experience amongst girls and IPV perpetration amongst boys) involved comparing incidence between the three study arms. An incident case of IPV or non-partner rape was one reported by a learner at the 12 months interview or, if not at 12, at 18 months. We did not include IPV responses at 6 months follow up as it was felt the time period was too short for a clear intervention effect and the IPV assessment measures asked about exposure to an (specific) act of violence ‘in the last 12 months’, so the question at 6 months included a pre-intervention period. We calculated person years of exposure as the time from baseline to the first report of IPV if the learner had dated at baseline, or half the time between the last report of not dating and the first report of dating plus time (if any) to the first report of IPV. We classified a learner as having ‘dated’ if they ever reported this, even if other information was contradictory. For learners remaining IPV free at the end of study, if dating throughout, person years of exposure was the time from baseline to the last interview.

Poisson regression modelling was used to compare incidence between the control arm and the two intervention arms, adjusting for clustering within schools and stratification by area. We considered baseline individual-level covariates for inclusion in the models: age, baseline IPV perpetration (boys)/experience(girls), race, family gender attitudes, childhood trauma experience, a learner’s school engagement score, alcohol or drug use and having ever had sex. We also included school-level summaries of the teacher’s perception of the school environment and teacher’s negative behaviour as covariates to account for any differences in environment among the schools. We fitted separate models for boys and girls Poisson regression modelling was also used to compare 12 months prevalence of non-partner violence experience/perpetration. The denominator for this analysis was all learners. Two sets of analysis were done for primary and secondary outcomes, one adjusted for honesty.

For secondary outcomes derived from scales, such as gender attitudes, communication and bullying, we calculated a score from scale items at each interview time point (baseline, 6 months, 12 months and 18 months). We used full maximum likelihood estimation to deal with missing total scores due to a missed follow up or due to a participant non-response. Prior to using full maximum likelihood estimation method, we examined patterns of missing item scores for each scale (outcome) at each time point. We also examined the proportion of missing item scores for each scale at each time point. The proportion of learners with missing item scores (partial missing or all missing) for the different scales ranged from <1% to 11%, with highest proportion of missing responses occurring at the 18m follow up. We also checked if there was any relationship between non-response to a scale and treatment arm, or with learner’s social demographic characteristics. As a sensitivity analysis, we imputed missing item scores using individual participant’s mean or using item mean adjusted for treatment arm. We then compared the overall mean scores (total score mean) between the 2 imputation methods and also against overall mean score with no imputation and found no significant difference in the scale means. With less than 15% of participants having missing total scores for the different outcome variables at the different time point, we preferred to use the full maximum likelihood method to account for missing data in the analysis.

Linear mixed effects models (multi-level model for change) were used to compare changes in continuous secondary outcomes over time between the control arms and the intervention arms [45]. The fixed effect terms included study arm, stratum, baseline covariates (as listed for the primary outcome) and time of interview, while the random effects were school with time point of interview as a covariate. The modelling also involved assessing any interaction between study arms and time. Kenward-Roger method was used to calculate denominator degrees of freedom in order to account for the small sample bias due to small number of clusters in each study arm [46]. We used a log-likelihood ratio test to compare any nested models such as a model with random intercept only against model with random intercept and random slope. Residual plots were done to check for model fit and normal distribution assumptions.

Logistic regression was used to compare binary outcomes such as condom use, engagement in transactional sex and use of contraceptives. The denominator for these binary outcomes was learners who had ever had sex. We compared incidence of pregnancy between arms using Poisson regression modelling, taking into account clustering by school. The exposure time was calculated the same way as in IPV and we used all learners as the denominator.

The analysis of the teachers’ data followed same procedures as in the learners in order to compare the intervention arms to the control arm. We also assessed the effect of positive discipline training on corporal punishment perpetration by teachers. For each outcome, we fitted a second model that adjusted for a teacher’s overall acknowledged honesty in responses but do not present this as the impact was negligible.

Analysis of data from caregivers involved fitting a random effects regression model for each outcome and measuring the slope of the line across the time points to assess the trend. Separate models were fitted for male and female caregivers. We calculated the mean at each time point for each continuous outcome.

## Results

**Fig 3:**
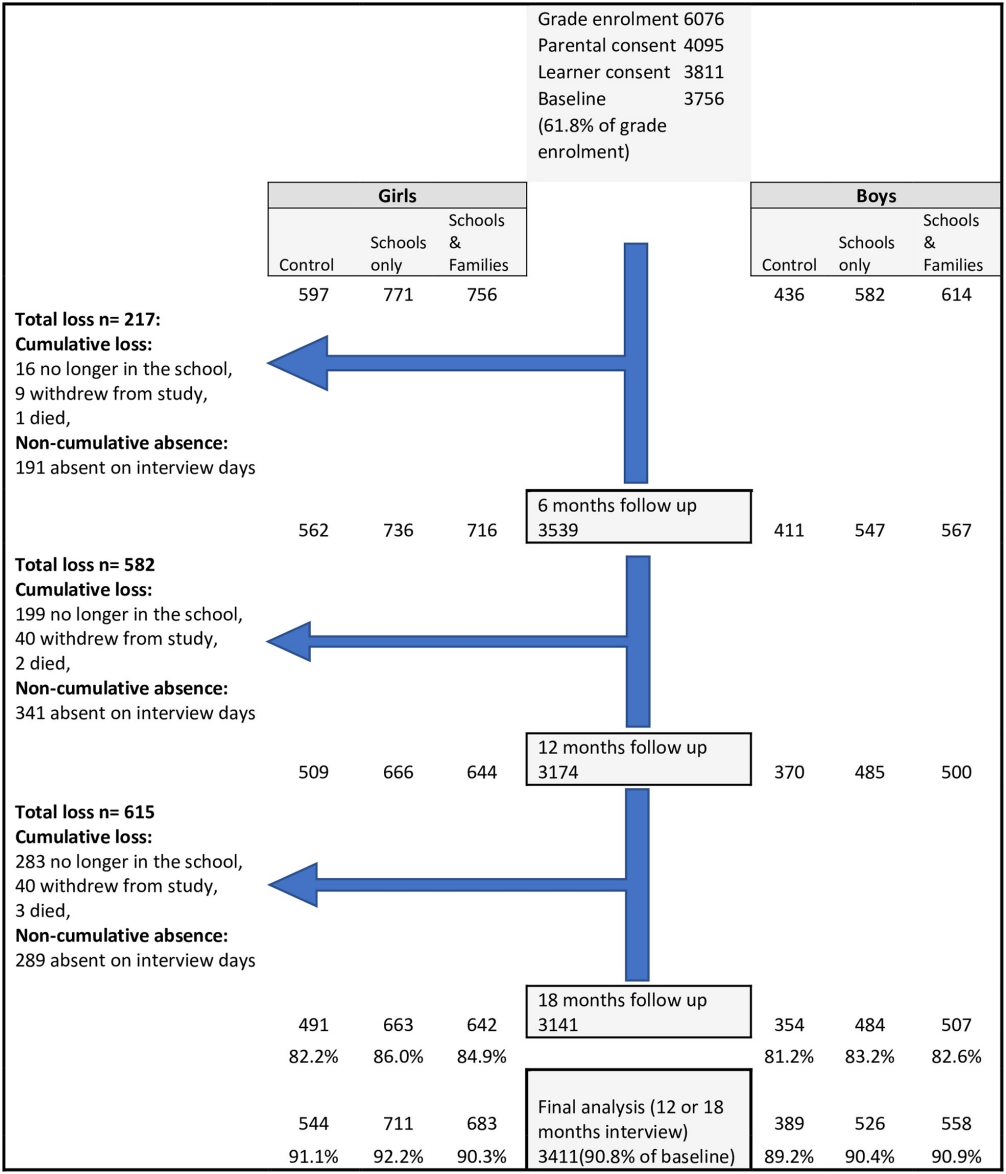
Trial profile.

The trial profile is shown in Fig 3. No schools (clusters) were lost to follow up. Twelve months follow up rates overall (across arms and genders) were 84.5% and at 18 months the overall follow up rate was 83.6%. There was little difference in follow up by arms (p = 0.47 for boys and p = 0.476 for girls). Loss to follow up was mainly because participants had moved home and could not be located. The main analyses for this study required pupils to have been enrolled at baseline and interviewed at 12 and/or 18 months follow up, and overall 90.8% of pupils were available for the final analysis. There were six serious adverse events among learners during the study. Three of the learners died, one was imprisoned, one was robbed of a cell phone at the school gate before an intervention session, and one child was reported to social workers due to parental alcohol abuse and child neglect. All deaths were from natural causes.

### Intervention attendance

In the schools that offered Skhokho for Families, there were 1376 children who were interviewed (of 2071 in Grade 8 in these schools) and 1144 of their caregivers agreed to participate in the project and themselves completed an interview. Of these, 562 (49.1%) caregivers attended the first session, 522 (45.6%) attended the second, 389 (34.0%) attended the third and 395 (34.5%) attended the fourth. Among the children, 549 (39.9%) attended the first, 566 (41.1%) attended the second, 459 (33.4%) attended the third and 458 (33.3%) attended the fourth. The school clubs were initially attended by 1007 pupils from the 16 schools where pupils were invited to attend (average 62.9 per school). 681 pupils attended the second session and 381 attended the tenth (and final) one (average 23.8 per school).

### Learners’ findings

Table 3 shows the learners’ baseline characteristics. As expected for Grade 8 in Tshwane schools, most participants were aged 12–15 years (2.7% of girls and 8.3% of boys were older than 15), were Black African (92% of girls, 90.8% boys) and many did not live with their biological caregivers, especially their fathers, and nearly one in five did not live with their mother. The population was fairly low income, with many male caregivers unemployed (affecting 28% of boys and 32.7% of girls). Female caregiver unemployment was even higher. Only two thirds of families lived in brick houses. Just under half of girls (47.3%) and a quarter of boys (28.9%) had a girlfriend or boyfriend and few girls (1.9%) but many more boys (17.6%) disclosed having ever had sex. The two arms were very similar, for both boys and girls, although the control arm girl participants were less likely to be Black African and more likely to live with their biological father.

**Table 3:** Social and demographic characteristics of participants in the three study arms.

	Girls			Boys		
	Control	Schools	Families &Schools	Control	Schools	Families &Schools
	n (%)	n (%)	n (%)	n (%)	n (%)	n (%)
**Age**						
12–13	323(54.3)	429(55.6)	470(62.1)	168(38.5)	246(42.2)	264(43)
14–15	244(41.0)	324(42.0)	276(36.5)	219(50.2)	290(49.7)	311(50.7)
16–19	28(4.7)	19(2.5)	11(1.5)	49(11.3)	47(8.1)	39(6.4)
**Race**						
African	526(88.3)	725(94.9)	703(93.4)	386(89.2)	541(93.6)	556(90.7)
Others	70(11.7)	39(5.1)	50(6.6)	47(10.8)	37(6.4)	57(9.3)
**Ever repeated a school year**	101(17.0)	116(15.1)	98(13.0)	131(30.3)	166(28.7)	159(26.0)
**Lives with biological mother**	489(82.1)	625(81.2)	617(81.9)	351(80.9)	468(80.8)	509(82.9)
**Lives with biological father**	320(53.7)	338(44.0)	351(46.7)	232(53.6)	289(49.9)	328(53.5)
**Male caregiver works**	398(66.9)	525(68.2)	506(67.2)	307(71.1)	427(73.6)	441(71.9)
**Main female caregiver works**	353(59.3)	465(60.4)	452(60.0)	257(59.1)	392(67.7)	377(61.4)
**Type of house**						
Brick	393(65.9)	559(72.8)	506(67.3)	275(63.2)	436(75.3)	418(68.1)
Wendy house /backyard dwelling	203(34.1)	209(27.2)	246(32.7)	160(36.8)	143(24.7)	196(31.9)
**Has a girlfriend or boyfriend**	304(51.2)	330(42.9)	371(49.5)	150(34.6)	153(26.4)	169(28.1)
**Has ever had sex**	12(4.1)	12(2.7)	18(4.8)	76(26.9)	101(23.7)	111(25.6)

There was no association between honesty and treatment group (p = 0.459 for girls and p = 0.206 for boys), however honesty of girls and boys differed, with 89.5% of girls and 84.1% of boys in the control arm asserting that they were honest, 91.6% of girls and 80.6% of boys in the schools arm and 90.3% of girls and 79.6% of boys in the families arm. For girls and boys, honesty declined with increasing age (in both cases p<0.001) (data not shown).

Table 4 shows the results for the comparison of incidence rates of physical or sexual IPV and non-partner sexual violence between the three study arms. The incidence of physical or sexual IPV was higher in all measures in the control arm than in the intervention arms. For boys, for any IPV the incidence rate (IR) per 100 person years were (33.3,31.8,31.1) for control, schools and families arms respectively and for girls, they were (29.1,26.0,26.8). For severe IPV rates for boys they were (26.3, 24.7, 26.1), and for girls (18.7, 15.8, 17.2). The adjusted incidence rate ratios were in all cases less than 1.00 but none were statistically significant. For boys, for any IPV the IRR for the school’s arm was 0.92 (CI: 0.72–1.17) and for the family’s arm IRR 0.90 (CI:0.71–1.13). For girls, for any IPV the IRR for the school’s arm was 0.84 (CI: 0.66–1.07) and for the families arm 0.88 (CI: 0.70–1.11). For severe IPV, for boys the IRR for the school’s arm was 0.94 (CI:0.72–1.23) and 0.98 (0.76–1.25) for the family’s arm. For girls in the schools arm it was 0.89 (CI: 0.64–1.23) and 0.98 (CI:0.71–1.34) for the family’s arm.

**Table 4:** Incidence of physical/sexual IPV and non-partner sexual violence perpetration/experience amongst learners who had ever dated.

	Outcome	Study arm	N	# of events	Rate per 100 person yrs	MODEL 1				MODEL 2			
						Adjusted incidence rate ratio*	95% CI			Adjusted incidence rate ratio*	95% CI		P value
							LCL	UCL	P value		LCL	UCL	
**Boys**	any IPV	Control	358	143	33.3								
		Schools	492	201	31.8	0.94	0.73	1.20	0.600	0.92	0.720	1.17	0.481
		Families	515	205	31.1	0.92	0.72	1.16	0.472	0.90	0.713	1.13	0.353
	Severe IPV	Control	358	113	26.3								
		Schools	492	156	24.7	0.96	0.73	1.27	0.770	0.94	0.72	1.23	0.641
		Families	515	172	26.1	1.00	0.77	1.30	0.997	0.98	0.76	1.25	0.861
	Non-partner violence	Control	389	99	17.6								
		Schools	526	140	18.2	1.0	0.75	1.32	0.980	0.99	0.74	1.31	0.928
		Families	558	140	17.3	0.98	0.75	1.28	0.873	0.96	0.74	1.26	0.786
**Girls**	any IPV	Control	453	148	29.1								
		Schools	624	195	26.0	0.83	0.66	1.06	0.131	0.84	0.66	1.07	0.159
		Families	593	184	26.8	0.88	0.70	1.11	0.285	0.88	0.70	1.11	0.282
	Severe IPV	Control	453	95	18.7								
		Schools	624	119	15.8	0.87	0.619	1.21	0.394	0.89	0.64	1.23	0.476
		Families	593	118	17.2	0.98	0.709	1.35	0.897	0.98	0.71	1.34	0.891
	Non-partner violence	Control	544	96	12.3								
		Schools	711	118	11.3	0.90	0.67	1.22	0.504	0.94	0.69	1.27	0.679
		Families	683	97	9.7	0.82	0.60	1.11	0.192	0.84	0.62	1.14	0.255

Adjusted incidence rate ratio*

Model 1: adjusted for baseline covariates (violence perpetration/experience, childhood trauma, age of learner, ever-had sex, teacher’s perception of school environment and teacher’s negative behaviour).

Model 2: adjusted for baseline covariates (violence perpetration/experience, childhood trauma, age of learner, ever-had sex, teacher’s perception of school environment and teacher’s negative behaviour) and for honesty in responding to questionnaire.

IPV analysis is among learners who have dated, non-partner rape analysis is among all learners.

For non-partner rape, for boys the IR was 17.6 in control, 18.2 in schools and 17.3 in the families arm, with the IRR 0.99 (CI:0.74–1.31) for the schools arm 0.96 (CI:0.74–1.26) for the families arm. For girls the IRs were 12.3, 11.3 and 9.7 respectively, with the IRRs 0.94(CI:0.69–1.27) and IRR 0.84 (CI:0.62–1.14) for the schools and the family’s arm.

Table 5 shows the results of the analysis of the other outcomes for boys. Less childhood trauma was reported among boys in the schools arm compared to the control arm with an Estimated Mean Difference (EMD) -0.73 (95%CI -1.41, -0.04, p = 0.037)) and some evidence of change in the same direction in the families arm (EMD = -0.13 (95%CI: -0.81–0.55, p = 0.685). Bullying was lower in the group with the schools intervention (EMD = -0.52 (95%CI -0.96, 0.08) p = 0.022)) and the effect was in the same direction in the families arm (EMD = -0.30 (95%CI -0.74, 0.14, p = 0.18)). The gender attitudes score improved across the intervention arms and the difference was statistically significant in the schools arm (EMD = 0.82 (95% CI 0.24, 1.39) p = 0.007)) and in the same direction in the families arm (EMD = 0.34 (95%CI -0.23, 0.92 p = 0.236). There were two measures of communication and in each case a higher score would denote better communication. The general direction was of better communication, and for boys in the school’s arm this was significantly better than the control arm boys (p = 0.04).

**Table 5:** Results of other outcomes for boys using linear mixed effect model.

	N	MODEL 1				MODEL 2			
		EMD	LCL	UCL	P value	EMD	LCL	UCL	P value
**Caregiver child communication scores (high = more communication)**									
Schools	526	0.21	-0.34	0.75	0.459	0.21	-0.33	0.76	0.435
Families	558	0.15	-0.39	0.69	0.588	0.62	-0.40	0.68	0.622
**Childhood trauma scores (high = more trauma)**									
Schools	526	-0.68	-1.38	0.01	0.054	-0.73	-1.41	-0.04	0.037
Families	558	-0.09	-0.78	0.61	0.798	-0.13	-0.81	0.55	0.695
**Bullying at school scores (high = more bullying)**									
Schools	526	-0.49	-0.95	-0.04	0.034	-0.52	-0.96	-0.08	0.022
Families	558	-0.25	-0.70	0.21	0.277	-0.30	-0.74	0.14	0.18
**Communication with girlfriend (high = more communication)**									
Schools	492	0.93	0.31	1.54	0.003	0.91	0.29	1.52	0.04
Families	515	0.18	-0.41	0.79	0.566	0.17	-0.44	0.79	0.58
**Delinquency scores (high = more delinquent)**									
Schools	526	0.15	-0.25	0.54	0.453	0.13	-0.27	0.52	0.520
Families	558	0.23	-0.17	0.62	0.250	0.21	-0.18	0.61	0.279
**Depression scores (high = more depressed)**									
Schools	526	-0.24	-1.23	0.75	0.615	-0.31	-1.27	0.64	0.501
Families	558	0.37	-0.62	1.36	0.448	0.30	-0.65	1.26	0.517
**Gender attitudes scores (high = more equitable)**									
Schools	526	0.76	0.15	1.37	0.016	0.82	0.24	1.39	0.007
Families	558	0.28	-0.33	0.89	0.359	0.34	-0.23	0.92	0.236

EMD = Estimated Mean Difference (relative to the control arm).

Model 1: adjusted for baseline covariates (age of learner and teacher’s perception of school environment) and time effect. Model 2: adjusted for baseline covariates (age of learner and teacher’s perception of school environment), time effect and honesty in responding to questionnaire.

Table 6 shows the results of the analysis of the other outcomes for girls. There was a reduction in reported bullying in the anticipated direction for both intervention arms, EMD = -0.23(95%CI -0.56, 0.10 p = 0.159) for schools and EMD = -0.34(95%CI -0.67, -0.01 p = 0.042) for the families arm. There was also a reduction in depression EMD = -0.774 (95%CI-1.50- -0.05, p = 0.037) for the school’s arm and EMD = -0.64 (95%CI -1.36–0.091 p = 0.083) for the families arm. As for the boys, the communication measures indicated change in the direction of better communication.

**Table 6:** Results of other outcomes for girls using linear mixed effect model.

	N	MODEL 1				MODEL 2			
		EMD	LCL	UCL	P value	EMD	LCL	UCL	P value
**Caregiver child communication scores (high = more communication)**									
Schools	711	0.25	-0.24	0.74	0.312	0.25	-0.23	0.74	0.310
Families	683	0.16	-0.34	0.65	0.531	0.54	-0.34	0.64	0.541
**Childhood trauma scores (high = more trauma)**									
Schools	711	0.08	-0.38	0.54	0.718	0.10	-0.35	0.56	0.647
Families	683	-0.03	-0.49	0.44	0.902	-0.02	-0.48	0.44	0.929
**Bullying at school scores (high = more bullying)**									
Schools	711	-0.26	-0.61	0.09	0.145	-0.23	-0.56	0.10	0.159
Families	683	-0.3	-0.69	0.02	0.063	-0.34	-0.67	-0.01	0.042
**Communication with boyfriend (high = more communication)**									
Schools	624	0.56	-0.03	1.16	0.064	0.57	-0.03	1.16	0.064
Families	593	0.40	-0.23	1.03	0.212	0.42	-0.21	1.05	0.192
**Delinquency scores (high = more delinquent)**									
Schools	711	-0.09	-0.37	0.18	0.489	-0.09	-0.36	0.18	0.510
Families	683	-0.21	-0.49	0.06	0.123	-0.21	-0.49	0.06	0.127
**Depression scores (high = more depressed)**									
Schools	711	-0.78	-1.50	-0.06	0.034	-0.77	-1.50	-0.05	0.037
Families	683	-0.63	-1.36	0.09	0.082	-0.64	-1.36	0.09	0.083
**Gender attitudes scores (high = more equitable)**									
Schools	711	0.17	-0.38	0.73	0.522	0.16	-0.33	0.66	0.501
Families	683	0.20	-0.36	0.76	0.466	0.23	-0.27	0.73	0.354

EMD = Estimated Mean Difference (relative to the control arm).

Model 1: adjusted for baseline covariates (age of learner and teacher’s perception of school environment) and time effect. Model 2: adjusted for baseline covariates (age of learner and teacher’s perception of school environment), time effect and honesty in responding to questionnaire.

Table 7 shows an analysis conducted among girls and boys who said they had ever had sex. The variables examined were condom use, contraceptive use and transactional sex, and girls were asked about pregnancy (reported at 12 or 18 months but not at baseline or 6 months). Reported condom use was higher among boys (aIRR 1.35 (95% Cl: 0.94–1.95) p = 0.107) and girls (aIRR 1.67 (95% Cl: 0.93–2.77), p = 0.087) in the school’s arm. In the family’s arm there was no evidence of impact among boys (aOR 0.98) but among girls the direction of effect also suggested more condom use (aOR 1.39 95% Cl 0.78, 2.48 p = 0.26). There was also an indication that contraceptive use was also higher among girls in the school’s arm (aIRR 1.64 95% Cl 0.95, 2.84, p = 0.079) and the families arm (aIRR 1.48 95% Cl 0.83, 2.65, p = 0.184). The consistent direction of effect was of a reduction in all intervention arm measures of transactional sex but all 95% Cls were wide. Ever having been pregnant was more often reported by 3.4% of girls in the school’s arm and 2.4% in the control arm (aIRR 1.88 95%CI 0.89, 4.00, p = 0.1). In the per protocol analysis, there was no transactional sex reported among the girls who attended the families workshops and their pregnancy rate was substantially lower than control girls (0.7% v. 2.4% aIRR 0.42 95% CI 0.09, 2.05 p = 0.28).

**Table 7:** Random effects Logistic/Poisson regression results for condom use, contraceptive us, transaction sex among learners, and incidences of pregnancy among girl learners.

Outcome	Study Arm	N	%‡	MODEL 1				MODEL 2			
				Adjusted OR/RRR	LCL	UCL	P value	Adjusted OR/RRR	LCL	UCL	P value
**Condom use**											
Boys	Control	199	46.7								
	Schools	278	54.3	1.34	0.93	1.94	0.114	1.35	0.94	1.95	0.107
	Families	305	46.2	0.97	0.68	1.39	0.866	0.98	0.68	1.40	0.894
Girls	Control	91	44.0								
	Schools	136	55.9	1.58	0.92	2.71	0.097	1.61	0.93	2.77	0.087
	Families	104	51.9	1.40	0.79	2.48	0.248	1.39	0.78	2.48	0.26
**Contraceptive use**											
Boys	Control	199	52.3								
	Schools	278	58.3	1.27	0.87	1.86	0.216	1.28	0.87	1.86	0.209
	Families	305	49.2	0.87	0.60	1.27	0.474	0.88	0.60	1.27	0.485
Girls	Control	91	47.3								
	Schools	136	59.6	1.61	0.94	2.77	0.083	1.64	0.95	2.84	0.079
	Families	104	56.7	1.49	0.84	2.64	0.173	1.48	0.83	2.65	0.184
**Transactional Sex**											
Boys	Control	199	12.1								
	Schools	278	9.6	0.84	0.43	1.63	0.607	0.82	0.42	1.57	0.54
	Families	305	10.6	0.77	0.40	1.48	0.424	0.74	0.39	1.42	0.362
Girls	Control	91	19.6								
	Schools	136	16.9	0.78	0.31	1.96	0.594	0.79	0.33	1.91	0.596
	Families	104	15.4	0.76	0.29	2.01	0.577	0.79	0.31	2.04	0.626
**Pregnancy**¥											
Girls	Control	544	2.4								
	Schools	711	3.4	1.86	0.86	4.03	0.113	1.88	0.89	4.00	0.100
	Families	683	2.2	1.36	0.59	3.15	0.476	1.34	0.59	3.06	0.489

^%‡^: Percentage of learner who used condom or contraceptives or who engaged in transactional sex, calculated as a percentage within each study arm of learner who had ever had sex.

^¥^: Effects (RRR) derived using Poisson regression.

Model 1: Adjusted for baseline covariates (age of learner, condom use/transactional sex/contraceptive use).

Model 2: Adjusted for baseline covariates (age of learner, condom use/transactional sex/contraceptive use) and honesty.

### Caregivers’ findings

S1 Table shows the characteristics of the caregivers who attended the Skhokho for Families intervention. Overall there were 857 women and 251 men who were interviewed at baseline. Most caregivers were aged between 30 and 49 years (S1 Table). Nearly half of the caregivers had completed high school themselves (Matric) or had tertiary education. Half the male caregivers (53.4%) were married compared to 34.5% of female caregivers. Three quarters of the caregivers were biological or stepparents and a quarter were grandparents or siblings. Overall 39% of children were orphaned (one or both caregivers). The families generally had very low income.

The change in health, parenting and gender attitudes are shown in Table 8 for caregivers who completed the last round of interviews. The analysis was also performed on data from all caregivers available for each interview time point and showed the same patterns (data not shown). From reports of the female and male caregivers, the impact of the intervention was very similar. There was evidence of sustained improvement in communication with their children (p = 0.12 for men and 0.043 for women), and sustained improvement in knowledge of the child (p<0.001 for both). There was a significant and sustained improvement in parenting stress (p = 0.014 for men and p = 0.004 for women). There was little change in the measure of positive parenting, but negative parenting significantly declined (improved) for women (p<0.001) and men (p = 0.003). There was some evidence of improvement in individual gender attitudes (p<0.001 for both) and improvement in perceptions of gender attitudes in the community (p = 0.012 for men and p<0.001 for women). There were also improvements in self-reported health (p<or = 0.001 for both). The overall measure of childhood trauma showed significant reduction of trauma over the 18 months (p<0.001 for both). There was a substantial reduction in the proportion of women who found it offensive talking about sex with their children (p<0.001), but little change among men.

**Table 8:** Parenting, health and gender attitudes (among those interviewed at baseline and round 5) in the school plus family’s arm.

	Pre-intervention		Post-intervention			p-value
	Baseline	Round 2	Round 3	Round 4	Round 5	
	Mean or %	Mean or %	Mean or %	Mean or %	Mean or %	
**MALE CAREGIVERS**	n = 124	n = 73	n = 111	n = 103	n = 124	
Communication with child (high = good)	18.4	18.3	18.4	18.5	19.2	0.120
Knowledge of child(high = good)	31.2	32.5	33.1	33.3	34.2	<0.001
Stress score (high = more stress)	21.7	20.7	19.5	19.9	19.2	0.014
Positive parenting score(high = good)	98.7	100.5	100.4	101.2	101.2	0.127
Negative parenting score(high = good)	19.8	20.4	20.1	20.8	20.8	0.003
Childhood trauma score (low = good)	12.6	11.1	11.3	10.9	10.5	<0.001
Individual gender attitudes (high = more equitable)	25.8	26.5	26.4	26.6	27.3	<0.001
Community gender attitudes (high = more equitable)	24.9	24.8	25.5	25.8	25.7	0.012
General Health score (high = good health)	12.7	13.3	13.7	13.9	13.8	<0.001
Embarrassed talking about sex with child (%)	35.6	31	28.6	30.9	29.8	0.497
Offended talking about sex with child (%)	33.9	31	28	30.9	29.8	0.672
**FEMALE CAREGIVERS**	n = 518	n = 319	n = 448	n = 446	n = 518	
Communication with child (high = good)	19.2	19.3	19.3	19.5	19.5	0.043
Knowledge of child(high = good)	31.6	32	33	33.6	34.2	<0.001
Stress score (high = more stress)	25	23.5	24.6	24.4	22.4	0.004
Positive parenting score(high = good)	99.6	100.5	100.7	100.7	100.3	0.629
Negative parenting score(high = good)	19.4	19.9	19.9	20.1	20.4	<0.001
Childhood trauma score (low = good)	13.3	12.5	12.2	11.8	11.8	<0.001
Individual gender attitudes (high = more equitable)	26.6	26.8	27.6	27.6	27.9	<0.001
Community gender attitudes (high = more equitable)	26	25.7	26.5	26.5	26.7	<0.001
General Health score (high = good health)	11.1	11.7	11.5	11.5	11.8	0.001
Embarrassed talking about sex with child (%)	27	30.5	24.7	25	26.3	0.290
Offended talking about sex with child (%)	34.5	33.9	29.1	29.6	24.7	<0.001

The reported intimate partner violence experience of female caregivers interviewed at round 5 is shown in Table 9 (the same pattern was seen for all female caregivers, data not shown). The women reported significantly less emotional IPV in the last 6 months across the time points (p<0.001), and thus less past 6 months IPV (p = 0.005), but the same was not seen for the other measures (Table 9).

**Table 9:** IPV experience by female caregivers (among those interviewed at baseline and round 5) in the family’s intervention.

	Pre-Intervention		Post-Intervention			p-value
	Baseline	Round 2	Round 3	Round 4	Round 5	
	%	%	%	%	%	
**In past 6m**	n = 371	n = 237	n = 336	n = 321	n = 388	
Emotional IPV	50.8	44.8	42.3	38.6	39.4	<0.001
Physical IPV	14.8	16.1	13.9	14.6	16.9	0.474
Sexual IPV	9.5	12.6	9.6	10.1	9.8	0.923
Any IPV	53.9	50.4	48.8	43.7	45.2	0.005

### Teachers’ findings

The three study arms were similar at baseline in the sex of teachers interviewed (proportion female: 41.4% in control, 39.4% in schools, 34% in families, p = 0.187), as well as the period they had worked at the school (mostly over 10 years) (p = 0.627). There was some evidence that there were more older teachers in the families arm as 36.5% of teachers were over 45 compared to 23.6% in the schools arm and 29.6% in the control arm, and the families arm had the fewest teachers under 30 (18%) compared to 32.4% in the schools arm and 27.2% in the control arm (p = 0.136).

Table 10 shows the mean scores at each time point for teachers’ perception of their school environment and challenges at school. At baseline the teachers in the control school had the most positive assessment of the school environment. By 18 months this somewhat declined but the environment in the schools’ arm and even more in the family’s arm schools was rated more positively. The test for the effectiveness of the interventions overtime relative to the control arm is shown in Table 10 and this shows that at 18 months teachers in both the schools and the families arms rated their school environment significantly better than that of the control schools. EMD = 1.41(95% Cl 0.15, 2.67 p = 0.029) for schools and EMD = 2.34(95% Cl 1.00, 3.68 p = 0.001) for the family’s arm.

**Table 10:** Teachers’ reports: Trends in mean scores or prevalence and effects over time.

		N	Mean/%	LCL	UCL	N	Mean/%	LCL	UCL	N	Mean/%	LCL	UCL	EMD#	LCL	UCL	p-value
Perception of school environment (high = good)	Control	169	55.9	54.9	57.0	141	56.0	54.8	57.1	135	54.1	52.6	55.6	Ref			
	School	259	53.5	52.6	54.4	200	53.8	52.8	54.8	191	53.4	52.3	54.5	1.41	0.15	2.67	0.029
	Families	200	53.9	52.8	54.9	138	55.6	54.4	56.9	153	55.6	54.4	56.7	2.34	1.00	3.68	0.001
Bullying in school (high = more bullying)	Control	169	35.1	33.8	36.3	141	35.2	33.8	36.6	135	34.3	32.8	35.7	Ref			
	School	259	34.9	33.9	35.8	200	34.2	33.1	35.3	191	34.1	33.0	35.2	0.59	-0.67	1.86	0.358
	Families	200	35.1	34.0	36.2	138	33.2	32	34.5	153	33.9	32.6	35.1	0.60	-0.75	1.96	0.382
Negative behavior (low = good)	Control	169	17.7	17.0	18.3	141	17.5	16.7	18.2	135	17.4	16.7	18.2	Ref			
	School	259	19.3	18.8	19.9	200	18.3	17.7	18.9	191	18.7	18.1	19.4	0.16	-0.57	0.88	0.670
	Families	200	18.7	18.0	19.3	138	18.4	17.7	19.1	153	17.7	17.0	18.4	-0.31	-1.09	0.46	0.424
Work Stress (high = more stress)	Control	169	24.1	22.4	25.7	141	24.2	22.3	26.1	135	26.3	24.1	28.4	Ref			
	School	259	23.7	22.5	24.9	200	23.7	22.3	25.2	191	25.7	24.0	27.4	-1.48	-3.33	0.36	0.116
	Families	200	25.0	23.3	26.6	138	23.9	22.1	25.7	153	25.7	23.8	27.7	-1.66	-3.63	0.32	0.100
Used corporal punishment‡	Control	169	6.5	3.7	11.3	141	7.8	4.4	13.6	135	7.4	4.0	13.3	Ref			
	School	259	14.3	10.6	19.0	200	9.0	5.7	13.9	191	7.9	4.8	12.7	-0.57	-1.72	0.57	0.328
	Families	200	13.5	9.4	18.9	138	10.1	6.1	16.4	153	6.5	3.6	11.7	-0.79	-2.04	0.45	0.213

^‡^ Percentage of teacher who used corporal punishment against learners. EMD = Estimated mean difference

^#^ Models adjusted for baseline scores, age of teacher and time effect. Effect measured at 18 months relative to the control arm.

Teachers in the control and the family’s arms assessed the problem of bullying in their schools at about the same level at baseline. At 12 and 18 months the problem of bullying was reported as lower in both schools and family’s arms but the trend was not statistically significant (p = 0.3 in both cases). Teachers self-reports of negative behaviour among teacher colleagues were significantly fewer in the control arm than the school’s arm at baseline and there was some evidence that they were better than in the family’s arm. There was a trend towards lower scores in all schools, but no statistically significant change.

Self-reported work stress was similar across schools at baseline and was higher in all schools at the 18 months measure, but the overall trend was of a reduction in reported stress over time in the intervention schools (p = 0.12 for schools arm and p = 0.10 for the families arm). At baseline 6.5% of teachers in the control arm admitted having used corporal punishment in the last 6 months, compared to 14.3% of the school’s arm and 13.5% of the family’s arm teachers. The trends in reduction overtime were in the direction of lower corporal punishment in intervention schools but the effects were not statistically significant. Greater effects were seen in an analysis adjusted for attendance at the positive discipline training but these were still not significant (schools arm EMD = -0.95 (95%CI -2.19, 0.29 p = 0.132) and the, families arm EMD = -1.10 (95%CI-2.40,0.20, p = 0.097).

## Discussion

This was a pragmatic randomised controlled trial and it was substantially under-powered. The planned number of schools were enrolled but the number of children per class who contributed to the primary outcome analysis being much lower than intended. Thus, caution is needed in interpreting results and the consistency and direction of effect is more informative than the actual tests of statistical significance, but the findings have considerable generalisability to a ‘real life’ teaching situation. The primary outcome for this study was IPV incidence and we did not demonstrate that exposure to the interventions resulted in significantly lower IPV incidence, however across all the measures of IPV there was a trend of lower IPV (whether any or severe) per intervention arm compared to the control arm. The same was seen for non-partner rape perpetration. Generally, the adjusted incidence rate ratios were lower for girls than boys and those measures for which this was the lowest, any IPV experience and non-partner rape, suggest the protection may have been in excess of 20%.

The central question in interpreting the findings is: did the interventions work? An easy answer would be that without statistically significant findings we must conclude that they did not. We are aware that not all interventions used in schools are effective and some research from the USA has shown than environment measures (restraining orders, posters and a security presence) may be better than a very brief classroom based intervention [20]. However, our study was substantially under-powered and so we need to be cautious about overly relying on p≤0.05 in interpreting the findings. We have rather pulled together a range of different pieces of information which we suggest point in the direction of benefit from the intervention. This does not preclude the possibility that further adaptation of the interventions based on the experienced of delivery may strengthen their effects.

The evidence that there may have been benefit from the intervention is supported by the secondary outcomes, which show less childhood trauma, bullying and more equitable gender attitudes in the schools intervention arm among boys, and less depression and bullying among girls. There was also evidence of impact on sexual and reproductive health with some evidence to suggest higher condom and contraceptive use among girls, and among boys in the school’s arm. This was supported by findings of no transactional sex reported by girls attending the intervention in the family’s arm and fewer pregnancies.

The evidence of impact from the intervention is strengthened by the findings of the caregivers’ reports. Whilst these are a non-control comparison finding, they show improvement in communication and knowledge of their child, lower childhood trauma perpetration, more equitable gender attitudes and lower parenting stress and better health. Female caregivers also reported less exposure themselves to IPV. The findings of the teachers’ interventions showed a general perception of a better school environment in the intervention schools. Taken together these results suggest that the Skhokho intervention is promising and deserves further research in a trial which is appropriately powered.

The incidence rates of violence reported by the dating Grade 8s seemed to be quite high and were much higher for boys than for girls. For girls, this is not out of keeping with other South African research on IPV, for example Russell et al found that 39% of dating Grade 8 girls in Cape Town had experienced physical IPV in the last 3 months [43]. However, the much higher prevalence of IPV and rape perpetration by boys than experience reported by girls is surprising. Although we asked about honesty and have adjusted analyses for self-reported honesty levels, we do not know how honest the children were in answering the honesty question. Thus, we may not have eliminated the problem of exaggeration of violence perpetration reporting by boys and this may account for the much lower degree of impact shown on the violence perpetration outcomes. The honesty adjustment did not make a very great difference, but we did note that the direction of benefit from the honesty adjustment was such that dishonest boys were exaggerating their use of violence against girls and women. This very likely reflects perceived social pressure on this age group of boys to demonstrate that they are in control of their girlfriends, a masculinity described among other male youth [47].

Change was seen in several secondary outcomes for boys and girls, but not across all of them or all intervention arms. There was a difference in the boys’ and girls’ assessment of communication, which were in the right direction by with no significant change shown, and caregivers’ own perceptions of improved communication. The difference may be explained by the fact that only 33–40% of the children in the family’s arm analysis attended the intervention, whereas most of the caregivers had attended some of it. The finding of a reduction in bullying in both boys and girls is important as bullying is a key risk factor of IPV and non-partner rape perpetration, found in this study and reported elsewhere [41, 48]. As is the reduction in depression in girls as this is another sign supporting the overall emerging picture suggesting that there may have been real change due to the interventions.

The main strength of this study is that it was a randomised controlled trial of a holistic school’s intervention with some evidence of impact on boys’ and girls’ reports on a set of gender-based violence measures. There have been remarkably few randomised controlled trials conducted in Africa to evaluate whole school interventions to prevent violence. Our interventions had an impact on IPV experience and perpetration of a much more modest degree than the 20–40% reduction often found in violence prevention trials [17, 49]. However, if our impact on IPV reported by girls in the school’s intervention was to be a realistic result (and only study replication will confirm this), then our interventions could be very important. The cost of providing our LO workbook to Grade 8 classes is minimal, given that learners are provided with such materials anyway, and the cost of providing about 3 days of extra training to LO teachers is also very low and it is possible that it could be provided by changing the focus of courses that the teachers already attend. We suggest that for very little investment the schools intervention could be scaled up nationwide and across an entire national cohort of grade 8s a reduction in violence of the degree found in this study would amount to a very large number of incidents of violence averted. The impact on sexual health and depression and gender inequitable attitudes are also incredibly important across the schooling system. Mental health and gender attitudes are key drivers of IPV perpetration and experience and so a sustained improvement in these would result in longer term IPV reductions [50].

The family’s intervention seemed to particularly contribute to enhanced non-partner rape prevention in girls, better sexual and reproductive health and less bullying. It is more costly and complex to deliver, but clearly it met a perceived need in the community and potentially impacted caregivers’ well-being as well as that of other siblings in the home. Our findings suggest a real perceived need here and that it should be subject to further research. The finding that caregivers report greater impact from the families interventions than children was also a pattern seen in the Sinovuyo evaluation and needs to be better understood [31]. It is possible that this is partly due to measurement error being less for adult respondents.

The trial has weaknesses which may impact on the interpretation of the results. Randomisation occurred prior to recruitment. No schools declined to participate in the study because of their allocation, but it may have influenced individual (or related parental) consent to participate. School classes were smaller than we anticipated before collecting actual class numbers and in total a third of caregivers did not give consent and a few learners with parental consent did not themselves consent (or were not present for interviews). This substantially reduced the sample size for the study overall and rendered the study underpowered. The problem was also compounded by the small proportion of learners who were dating, and the fact that at this age ‘dating’ often means something very different from its meaning in older age groups [51]. In this context the interpretation of results probably more safely follow the principle used in interpreting the results of the SASA trial in Uganda, where looking for consistency and change in the anticipated direction was seen as particularly salient [44].

In the context of South Africa, we do not expect the lack of caregiver consent to be an indication of non-acceptability of the intervention or the research to caregivers, as generally researchers working with adults find few people who are asked to participate in research decline. It is more likely that the caregivers, in most cases, were not shown the consent form by the learners. This may reflect poor caregiver teenager communication, especially in the context of many not living with biological parents, and it may reflect learner apathy, which is also a problem in South African schools. Since the interventions were offered to the schools and learners in the two intervention arms irrespective of research participation, and the uptake of the caregiver teenager workshops was surprisingly high, and among those doing the interviews loss to follow up was very low, it is not very likely to have reflected unacceptability of the interventions or research process.

We have no way of knowing the extent to which there was fidelity to the classroom-based LO intervention, our design is therefore an assessment of the impact of making the materials available in schools rather than the impact of the LO materials themselves, which fits with the description of this as ‘pragmatic’. There is often a failure of schools to follow the national LO curriculum in general, with the syllabus often not finished and LO lessons commonly being skipped or re-directed to gain, for example, an extra maths period. Further by offering teacher training we noted that some teachers cycled in and out of LO teaching, even within a single school year, which is very disruptive. We also noticed that a number of teachers who had made a career teaching another subject were given the LO class in the months before their retirement.

The participation of caregivers in the Families workshops needs to be put in context. In South Africa there is a really little parental participation in normal public schools (there is more in the privileged public schools known as Model C schools which were not in our study). It is commonly said that at most 5% of caregivers attend meetings called by schools, although we are not sure of the origin of this statistic. We found that two thirds of invited caregivers were willing for their children to participate in our research and about half of these (which is about a third of all caregivers) came to the first workshop, and by the end just under a quarter completed all four workshops. Given that workshop participation had to compete with Saturday work, funerals, weddings and community events, family shopping and Saturday churches, this level of attendance seems pretty good. It is clear a testament to the importance caregivers give to addressing the problem of relationships with their children and the fact that attrition was not higher, speaks to the perceived value of the workshops. This is supported by (unpublished) qualitative data. Having said this, our families intervention was only delivered to between half and a third of learner research participants and their caregivers in the combined schools and families arm schools and this will have impacted on the ability of this intervention to show effect in the intervention arm. Unfortunately, our limited sample size has made meaningful sub-group analysis very difficult. The parents’ results are encouraging, but we had no control arm and are aware that results are always vulnerable to a Hawthorne effect. We were encouraged by the fact that we didn’t see a uniform change in all indicators, as hypothesised, as in real life this is rarely seen and the lack of uniformity gives greater confidence in the veracity of reporting and thus in the findings where change was detected.

We used dating as a denominator in the analysis of the main outcome (IPV), but are aware that South African research shows that some of those ‘dating’ may not be ‘dating’ as older adolescents and adults know it [51]. For example, ‘dating’ in this age group may mostly involve much texting and very little contact time, although even this may include threats and controlling behaviour. This may explain why some learners gave inconsistent answers over time to whether they had ever dated or not. We do not know if this was due to concealing dating, initial over-reporting of dating, or at some point perceiving oneself to ‘have a girlfriend or boyfriend’ when the relationship activities involved minimal contact, and then at another point in time reclassifying this as not having been in a girlfriend/boyfriend relationship. It should be noted that this did not apply to all, as others in the same year were sexually active and some had given birth. We classified a learner as having ‘dated’ if they ever reported this, which may have overestimated dating and impacted on the scope for change in some participants. We are also concerned about overreporting of having had sex by boys. In South African research boys generally report having sex younger than girls and adults are usually sceptical, and so the veracity of sexual activity reports of boys is uncertain[52] [53].

The generalisability of the study findings may be influenced by a number of aspects of the trial design. The interventions were delivered in the context of multi-component arrangements and it is hard to know if taking a single piece of this, for example just the Families intervention, would be beneficial on its own. It is our opinion from running them that the school clubs are unlikely to have contributed much to the overall intervention effect as so few children per school attended and this is likely to be below the level at which impact could be anticipated. This is why we have not discussed them much in the paper. We would recommend further evaluation of the interventions in contexts where it may be possible to monitor fidelity to the LO workbooks and also to evaluate the Families intervention outside the context of a multi-component study. The study findings are a measure of the difference in outcomes between the intervention and control arms. We cannot exclude the possibility that the normal LO curriculum resulted in behaviour change, although it would be surprising if the control intervention had a substantial impact.

Sample size calculations in future evaluations of school-based interventions to prevent IPV should use a more modest estimate of expected effect size, need to be mindful of the difficulties in getting parental consent and fully aware of the relatively low levels of conventional dating among 13–15 year olds. One positive recommendation from this study is that very large sample sizes are required to assess the modest but important reductions in IPV incidence that may result from behavioural interventions and the necessary funding should be provided.

There could have been contamination between arms, but serious contamination is unlikely as in this age group children predominantly mix with school friends or family. Our follow up rates were very high and follow up rates were very similar in the intervention and control arms this is unlikely to have biased the results. A weakness of the study is that we only had any follow up to 18 months post-baseline and the LO intervention was rolled out over the whole of the year from January–October 2014 and some of the clusters only finished the Families workshops in October 2014. Thus, the end-line was actually conducted 10–12 months post- the end of the intervention and this may not have been sufficient time for its full impact to be realised. In the literature on IPV prevention it is common for effect to only be seen two years after baseline [17, 44, 49]. It is possible the intervention effect would have been greater if there had been a further round of data collection. However, the trial was only funded for two years of research and we were concerned that we would have much more loss to follow up if we continued to track participants into Grade 10 as there is quite considerable drop out from South African schools at the end of Grade 9[54].

The study was not blinded. Obviously, the schools knew which arm they were allocated to, the pupils were aware of what they had been offered and the research team knew which arm schools were in. Data was collected through self-completed questionnaires, however, and so there was not likely researcher bias in data collection. It is impossible to remove any bias that could have been a result of students knowing the study arm they were in or bias in reporting due to use of repeated measures, in a study of this nature. We had some questions that were doubled barrelled and the time frame of reporting varied a little between questions. It’s very unlikely that this would have introduced differential bias between the arms.

The impact of the family’s intervention on caregivers is indicated by the trends of improved parenting and better reported own health. The reduction in women reported emotional IPV is also valuable. There was no control arm, so some caution is needed in interpreting findings, but the trend validity is strengthened by two baseline measures. The overall picture is of positive impact on families, which supports the reported impact on children.

### Conclusions

This under-powered pragmatic IPV reduction trial has shown evidence a generally beneficial impact of the Skhokho intervention on a number of outcome measures, when viewed by both adolescents and caregivers, and their teachers. The direction of effect overall is of reduced IPV and non-partner rape experience (for girls) and perpetration (for boys) for all measures and there were significant reductions in a number of IPV risk factors including bullying (reported by boys and girls), inequitable gender attitudes (boys) and depression (girls). As well as in indicators of sexual and reproductive health including condom use and contraception and in those attending the family’s intervention, transactional sex and pregnancy. These positive outcomes were supported by the changes reported by caregivers, including a reduction in female caregivers’ own IPV experiences, which is another risk factor for IPV among adolescents. The findings show promise which is similar to that seen in the evaluation of the intervention SASA [44]. Given the context of the intervention being highly scalable within South African schools at a relatively low cost, we suggest further research into Skhokho should be conducted and particular attention given in this to teacher training.

## Supporting information

S1 Table Characteristics of care-givers. (DOCX)

S1 File Questionnaire administered to learners. (XLSX)

S2 File Questionnaire administered to parents. (XLSX)

S3 File Questionnaire administered to teachers. (XLSX)
