# Supplementary material for: RCT evaluation of Skhokho: A holistic school intervention to prevent gender-based violence among South African Grade 8s
Source: PLoS One. 2019 Oct 29;14(10):e0223562. doi: 10.1371/journal.pone.0223562 (PMC6818771; doi:10.1371/journal.pone.0223562)
Supplement: S1 Table — (DOCX) [file pone.0223562.s001.docx]

| S2 Table : Characteristics of all caregivers enrolled for round one | | | | | | |
| --- | --- | --- | --- | --- | --- | --- |
|  | Total(N=1118) | | Male(N=251) | | Female(N=867) | |
|  | **n** | % | n | % | n | % |
| **Age of caregiver** |  |  |  |  |  |  |
| 20-29yrs | 130 | 11.6 | 29 | 11.6 | 101 | 11.7 |
| 30-39yrs | 367 | 32.8 | 53 | 21.1 | 314 | 36.2 |
| 40-49yrs | 387 | 34.6 | 105 | 41.8 | 282 | 32.5 |
| >=50yrs | 221 | 19.8 | 62 | 24.7 | 159 | 18.3 |
| missing | 13 | 1.2 | 2 | 0.8 | 11 | 1.3 |
| **Education level** |  |  |  |  |  |  |
| No school | 74 | 6.6 | 16 | 6.4 | 58 | 6.7 |
| Primary | 162 | 14.5 | 34 | 13.6 | 128 | 14.8 |
| Secondary | 368 | 32.9 | 79 | 31.5 | 289 | 33.3 |
| Matric | 317 | 28.4 | 73 | 29.1 | 244 | 28.1 |
| Tertiary | 197 | 17.6 | 49 | 19.5 | 148 | 17.1 |
| **Marital Status** |  |  |  |  |  |  |
| Married | 433 | 38.7 | 134 | 53.4 | 299 | 34.5 |
| Living together but not married | 208 | 18.6 | 53 | 21.1 | 155 | 17.9 |
| Not living together | 201 | 18.0 | 29 | 11.6 | 172 | 19.8 |
| No partner | 275 | 24.6 | 35 | 13.9 | 240 | 27.7 |
| missing | 1 | 0.1 |  |  | 1 | 0.1 |
| **Relationship with child** |  |  |  |  |  |  |
| Biological parent | 726 | 64.9 | 153 | 61.0 | 573 | 66.1 |
| Step or foster parent | 109 | 9.8 | 41 | 16.3 | 68 | 7.8 |
| Granny/Uncle/Aunt | 169 | 15.1 | 24 | 9.6 | 145 | 16.7 |
| Brother/sister | 113 | 10.1 | 33 | 13.2 | 80 | 9.2 |
| missing | 1 | 0.1 |  |  | 1 | 0.1 |
| **Status of Biological parents** |  |  |  |  |  |  |
| Mother alive | 294 | 26.3 | 42 | 16.7 | 252 | 29.1 |
| Father alive | 66 | 5.9 | 18 | 7.2 | 48 | 5.5 |
| Both parents alive | 680 | 60.8 | 175 | 69.7 | 505 | 58.3 |
| None alive/ don’t know | 78 | 7.0 | 16 | 6.4 | 62 | 7.2 |
| **Monthly income** |  |  |  |  |  |  |
| <=R500 | 406 | 36.3 | 69 | 27.5 | 337 | 38.9 |
| R501-R1000 | 239 | 21.4 | 35 | 13.9 | 204 | 23.5 |
| R1001-R2000 | 198 | 17.7 | 37 | 14.7 | 161 | 18.6 |
| R2001-R5000 | 160 | 14.3 | 59 | 23.5 | 101 | 11.7 |
| >R5000 | 101 | 9.0 | 49 | 19.5 | 52 | 6.0 |
| missing | 14 | 1.3 | 2 | 0.8 | 12 | 1.4 |
